# Overcoming challenges to discover drugs for liver stages of *Plasmodium vivax*

**DOI:** 10.1186/1475-2875-13-S1-O23

**Published:** 2014-09-22

**Authors:** Dennis E Kyle

**Affiliations:** 1 Department of Global Health, College of Public Health, University of South Florida, USA

## 

Perhaps the most daunting challenge for eliminating malaria is developing novel interventions to eradicate the dormant hypnozoite of *Plasmodium vivax*. New *in vitro* models are urgently needed to accelerate drug and vaccine discovery for liver stages of *P. vivax* malaria, yet maintaining the physiology of primary hepatocytes in long-term culture *in vitro* remains a major obstacle. Several advanced liver models support hepatic phenotypes necessary for drug and disease studies, yet these models are characterized by intricate features such as co-culture with one of more supporting cell types or advanced media perfusion systems. Regardless of the culture system, primary hepatocyte culture systems suffer from reproducibility issues due to phenotypic variation and expensive, limited supplies of donor lots. We have developed a microfluidic bilayer device that sustains primary human hepatocyte phenotypes, including albumin production, factor IX production, cytochrome P450 3A4 drug metabolism and bile canaliculi formation for weeks in a simple monoculture format with static media. Using a variety of channel architectures, we discovered how primary cell phenotype is promoted by spatial confinement within the microfluidic channel, without the need for perfusion or co-culture. This new model is amenable to 384 well screening platforms and utilizes a few hundred primary human hepatocytes, maintains hepatocyte function for weeks *in vitro* within a relatively simple model, and addresses many of the major hurdles in human hepatocyte culture research.

